# LPS Triggers Acute Neuroinflammation and Parkinsonism Involving NLRP3 Inflammasome Pathway and Mitochondrial CI Dysfunction in the Rat

**DOI:** 10.3390/ijms24054628

**Published:** 2023-02-27

**Authors:** Irais E. Valenzuela-Arzeta, Luis O. Soto-Rojas, Yazmin M. Flores-Martinez, Karen M. Delgado-Minjares, Bismark Gatica-Garcia, Juan U. Mascotte-Cruz, Porfirio Nava, Omar Emiliano Aparicio-Trejo, David Reyes-Corona, Irma A. Martínez-Dávila, M. E. Gutierrez-Castillo, Armando J. Espadas-Alvarez, Carlos E. Orozco-Barrios, Daniel Martinez-Fong

**Affiliations:** 1 Departamento de Fisiología, Biofísica y Neurociencias, Centro de Investigación y de Estudios Avanzados del Instituto Politécnico Nacional, Mexico City 07360, Mexico; 2 Laboratorio de Patogénesis Molecular, Laboratorio 4 Edificio A4, Carrera Médico Cirujano, Facultad de Estudios Superiores Iztacala, Universidad Nacional Autónoma de México, Mexico City 54090, Mexico; 3 Red MEDICI, Carrera Médico Cirujano, Facultad de Estudios Superiores Iztacala, Universidad Autónoma de México, Mexico City 54090, Mexico; 4 Programa Institucional de Biomedicina Molecular, Escuela Nacional de Medicina y Homeopatía, Instituto Politécnico Nacional, Mexico City 07320, Mexico; 5 Nanoparticle Therapy Institute, Aguascalientes 20120, Mexico; 6 Departamento de Fisiopatología Cardio-Renal, Instituto Nacional de Cardiología Ignacio Chávez, Mexico City 14080, Mexico; 7 Departamento de Biociencias e Ingeniería, Centro Interdisciplinario de Investigaciones y Estudios Sobre Medio Ambiente y Desarrollo, Instituto Politécnico Nacional, Mexico City 07340, Mexico; 8 Conacyt-Unidad de Investigaciones Médicas en Enfermedades Neurológicas, Hospital de Especialidades Dr. Bernardo Sepúlveda, Centro Médico Nacional Siglo XXI, Instituto Mexicano del Seguro Social, Mexico City 06720, Mexico; 9 Programa de Nanociencias y Nanotecnología, CINVESTAV, Mexico City 07360, Mexico

**Keywords:** neuroinflammation, Parkinson’s disease, senescence, motor behavior deficits, neurotoxic A1 astrocytes, neurotrophic A2 astrocytes, caspase-1

## Abstract

Whether neuroinflammation leads to dopaminergic nigrostriatal system neurodegeneration is controversial. We addressed this issue by inducing acute neuroinflammation in the substantia nigra (SN) with a single local administration (5 µg/2 µL saline solution) of lipopolysaccharide (LPS). Neuroinflammatory variables were assessed from 48 h to 30 days after the injury by immunostaining for activated microglia (Iba-1 +), neurotoxic A1 astrocytes (C3 + and GFAP +), and active caspase-1. We also evaluated NLRP3 activation and Il-1β levels by western blot and mitochondrial complex I (CI) activity. Fever and sickness behavior was assessed for 24 h, and motor behavior deficits were followed up until day 30. On this day, we evaluated the cellular senescence marker β-galactosidase (β-Gal) in the SN and tyrosine hydroxylase (TH) in the SN and striatum. After LPS injection, Iba-1 (+), C3 (+), and S100A10 (+) cells were maximally present at 48 h and reached basal levels on day 30. NLRP3 activation occurred at 24 h and was followed by a rise of active caspase-1 (+), Il-1β, and decreased mitochondrial CI activity until 48 h. A significant loss of nigral TH (+) cells and striatal terminals was associated with motor deficits on day 30. The remaining TH (+) cells were β-Gal (+), suggesting senescent dopaminergic neurons. All the histopathological changes also appeared on the contralateral side. Our results show that unilaterally LPS-induced neuroinflammation can cause bilateral neurodegeneration of the nigrostriatal dopaminergic system and are relevant for understanding Parkinson’s disease (PD) neuropathology.

## 1. Introduction

Recent experimental and clinical findings support that neuroinflammation plays a pivotal role in Parkinson’s disease (PD) [1], which is the second most common neurodegenerative illness worldwide, characterized by motor and non-motor dysfunctions [2,3]. Furthermore, neuroinflammation can be the primary event of dopaminergic neurodegeneration in idiopathic parkinsonism [4], triggering pathological mechanisms of cellular senescence, oxidative stress, microglial cell activation, and neurotoxic reactive astrocyte induction [5,6,7]. In addition, the enriched presence of microglial cells in the *substantia nigra* (SN) and surrounding brain regions contributes to the increased vulnerability of dopaminergic neurons to neuroinflammation [8,9]. Other aggravating factors include the high concentration of oxidized dopamine products [10], the increased level of neuromelanin-associated redox-active iron [11], the low levels of glutathione (GSH), and decreased glutamylcysteine ligase activity in the substantia nigra (SN) [12].

The consensus in the literature is that astrocytes play a dual role in neurodegenerative diseases, restraining the inflammatory process on one side and inducing neuronal death on the other [13]. Transcriptome analyses have correlated these opposite functions with specific phenotypes of reactive astrocytes, the neurotoxic A1 type, identified by complement component 3 (C3) expression, and the anti-inflammatory A2 type, revealed by S100 calcium-binding protein A10 (S100A10) expression [5]. Activated microglia are responsible for inducing the neurotoxic A1 phenotype in astrocytes by releasing interleukin-1 alpha (IL-1α), tumor necrosis factor-alpha (TNF-α), and the complement component subunit 1q (C1q) [5,14,15]. Neurotoxic A1 astrocytes trigger neuronal death by releasing still-unknown molecules. By contrast, the induction of A2 reactive astrocytes by prokineticin-2 (PK2) leads to a decrease in pro-inflammatory factors, an upregulation of the antioxidant genes *arginase-1* and *nuclear factor erythroid 2-related factor 2* (*Nrf2*), and of the high-affinity glutamate/aspartate transporter (GLAST), the latter leading to an increase in glutamate uptake [16]. In addition, A2 reactive astrocytes release vascular endothelial growth factor (VEGF), brain-derived neurotrophic factor (BDNF), and nerve growth factor (NFG) [17], which promote neural survival, axonal growth, remyelination in a cuprizone-induced model [18], and synaptic repair [19].

Microglial cells have long been considered the professional immune cells of the central nervous system whose activation unfailingly leads to neuroinflammation in neurodegenerative diseases [20]. The importance of microglial cells in PD stems from their direct action in eliminating vulnerable dopaminergic neurons via pro-inflammatory cytokines, oxidants, nitric oxide, and phagocytosis as part of an innate immune response [21,22] but also from their indirect action inducing neurotoxic astrocytes A1 and attracting professional immune cells; the latter response involves the participation of the adaptive immune system [23]. Furthermore, microglial activation constitutes the basis for the endotoxin hypothesis of neurodegeneration [24] because lipopolysaccharide (LPS), the classic bacterially-derived endotoxin, activates microglial cells directly through the Toll-like receptor 4 (TLR4) [25]. The crucial role of microglia in neurodegeneration is the target of new antiparkinsonian treatments aiming to prevent microglial activation [26,27] or block the microglia-induced A1 astrocyte conversion [28]. The unique feature of microglia is that changes in the cell form reflect their activation state and function in the SN [7,29,30], thus facilitating the microglia evaluation by immunoreactivity for ionized calcium-binding adapter molecule 1 (Iba1) [7,31,32].

Microglia and astrocytes express inflammasome 3 (NLRP3), a subcellular multiprotein complex whose activation by infectious agents and host-derived molecules mediates caspase-1 activation [33,34]. This evolutionarily conserved enzyme proteolytically cleaves the precursor interleukin 1β (IL-1 β) to an active secreted molecule that can cause neuronal damage through various downstream signaling pathways [35]. Furthermore, recent evidence shows that NLRP3-activating stimulations induce mitochondrial dysfunction by disrupting mitochondrial potential and mitochondrial complex-I (CI) activity [36]. Therefore, the evaluation of microglial activation/A1 reactive astrocytes and their association with the NLRP3 inflammasome multiprotein complex activation and mitochondrial IC dysfunction is critical to understanding the mechanisms of LPS-induced neuroinflammation to cause neurodegeneration of the dopaminergic nigrostriatal system.

Previous work from our group showed that a single microdose of LPS into the SN acutely elicits microglia activation and the release of nitric oxide and pro-inflammatory cytokines (TNF-α, IL1-β, and IL-6), followed by increasing reactive astrocytes and infiltrating lymphocytes [29]. Since the loss of tyrosine hydroxylase (TH) positive neurons was not statistically significant at early times (1, 2, 3, 5, 8, 24, 48 h) but only on day seven after LPS administration, which was the endpoint of that study, we hypothesized that neurodegeneration of the nigrostriatal dopaminergic system would occur later (30 days after the LPS insult) [29]. Therefore, the present study aimed to identify the phenotype (A1 and A2) of reactive astrocytes and microglia activation and its association with both NLRP-3 inflammasome activation and mitochondrial complex I dysfunction in the LPS-induced acute neuroinflammation model. In addition, we extended the study to 30 days after LPS administration to assess the senescence of dopaminergic neurons, characterize the progressive neurodegeneration of the nigrostriatal system, and observe the resulting motor deficits in experimental animals. Our results support the endotoxin hypothesis of neurodegeneration [24], at least for the particular case of PD.

## 2. Results

### 2.1. Intranigral Injection of LPS Elicits Fever and Sickness Behavior in Rats

**Figure 1:**
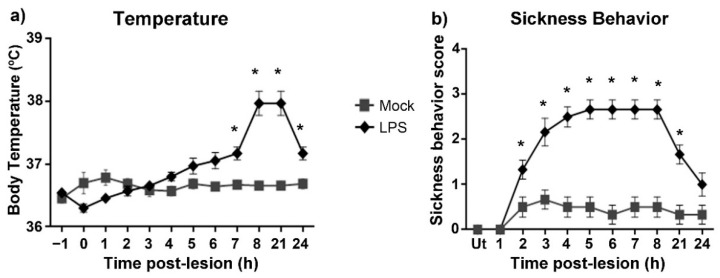
Clinical evolution after a single injection of LPS in the left SN of the rat. (**a**) Body temperature and (**b**) Sickness behavior score post-lesion. Mock = rats injected with the vehicle (2 μL of endotoxin-free physiological saline solution) in the left SN. Ut = Untreated rats. All values represent the mean ± standard error of the mean (SEM) (*n* = 6 rats). Asterisks (*) denote differences (*p* < 0.05) between the LPS and Mock groups. Repeated-measures two-way ANOVA and Tukey post hoc test were applied.

LPS aroused acute and reversible infection-like signs when intranigrally injected in rats. In the Mock group, body temperature remained constant at 36.7 ± 0.6 °C during the evaluation period. In contrast, increasing body temperature was detected from 4 h post-injection, reaching a maximum (37.95 ± 0.75 °C) between 8 and 21 h (*p* < 0.05) and decreasing to 37.1 ± 0.3 °C at 24 h (Figure 1a).

Rats with LPS intranigral injection developed sickness behavior from 3 h post-injection, reaching a plateau at 5 h and then decreasing at 21 h (*p* < 0.05; Figure 1b). Between 3 and 8 h after LPS injection, the main signs of sickness were adynamia, curled body posture, closed eyes, and piloerection; the latter response remained until the end of measurements (Figure 1b). Mock rats did not develop sickness behavior (*p* > 0.05), except adynamia in 20% and irregular fur in 15% of the Mock group compared with the Ut control group (Figure 1b).

### 2.2. Microglia Activation after LPS Intranigral Injection

**Figure 2:**
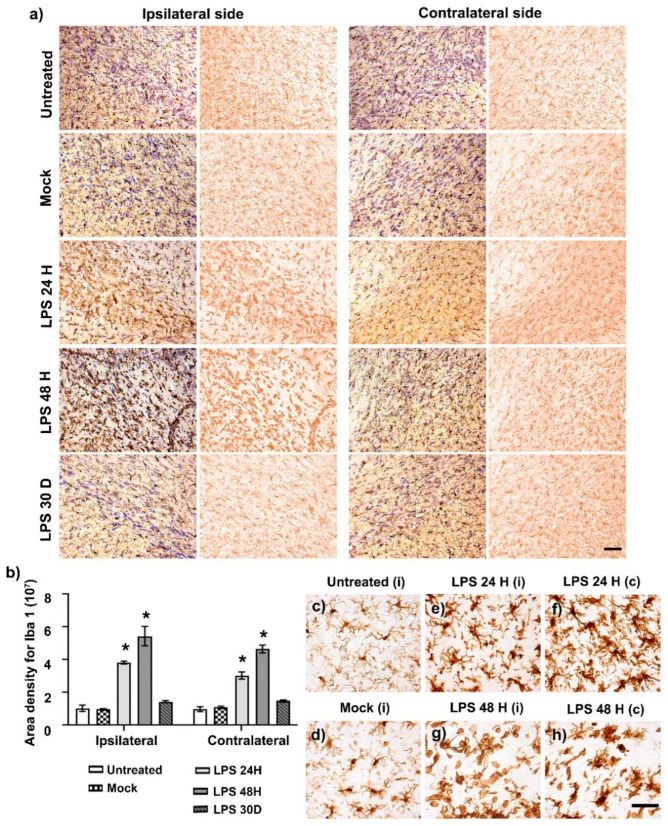
Activation and polymorphism of microglial cells in the SNpc after LPS injection. (**a**) Representative micrographs of Iba 1 (+) cells and Nissl counterstain in the SNpc of control groups (Ut and Mock) and groups at different times post-LPS injection (shown at the left margin in every row). (**b**) The graph shows the Iba1 area density measured on immunohistochemistry assays in (**a**) using ImageJ software v.1.46r (National Institutes of Health; Bethesda, MD, USA). The bars show the mean ± SEM calculated from the measurements in three anatomical levels of the same rats. *n* = 3 independent rats per group. The asterisk (*) compares the LPS group with the Mock group on both sides, *p* < 0.05. Two-way ANOVA and post hoc Tukey tests. Representative amplified micrographs show several microglial forms in the SNpc of the Ut (**c**) and Mock (**d**) groups to compare the activation state induced by LPS in the ipsilateral (**e**,**g**) and contralateral sides (**f**,**h**). The scale bars = 50 μm are for all micrographs.

As expected, intranigral LPS administration activated microglial cells on the injected side, evidenced by a 396% increase of Iba1 immunoreactivity at 24 h compared to the Mock group (*p* < 0.05), reaching a maximum response (563%) at 48 h (*p* < 0.05) and decreasing to reach basal values on day 30 after injection (*p* > 0.05). Interestingly, a statistically significant 277% increase (*p* < 0.05) of Iba1 (+) cells with a similar time course to the injected side also occurred on the contralateral side compared with the Mock group (Figure 2a,b). Indeed, there was no significant difference (*p* > 0.05) comparing the ipsilateral and contralateral responses. In addition, altered cell shapes were observed in LPS-treated animals compared with the quiescent cell forms observed in the ipsilateral side of the Ut (Figure 2c) and Mock groups (Figure 2d). At 24 h after LPS injection, reactive cell forms were predominantly observed in the ipsilateral (Figure 2e) and contralateral (Figure 2f) SNpc. At 48 h, amoeboid cells were more abundant in the ipsilateral SNpc (Figure 2g) than in the untreated side (Figure 2h).

### 2.3. LPS Preferentially Induces the Neurotoxic A1 Phenotype

**Figure 3:**
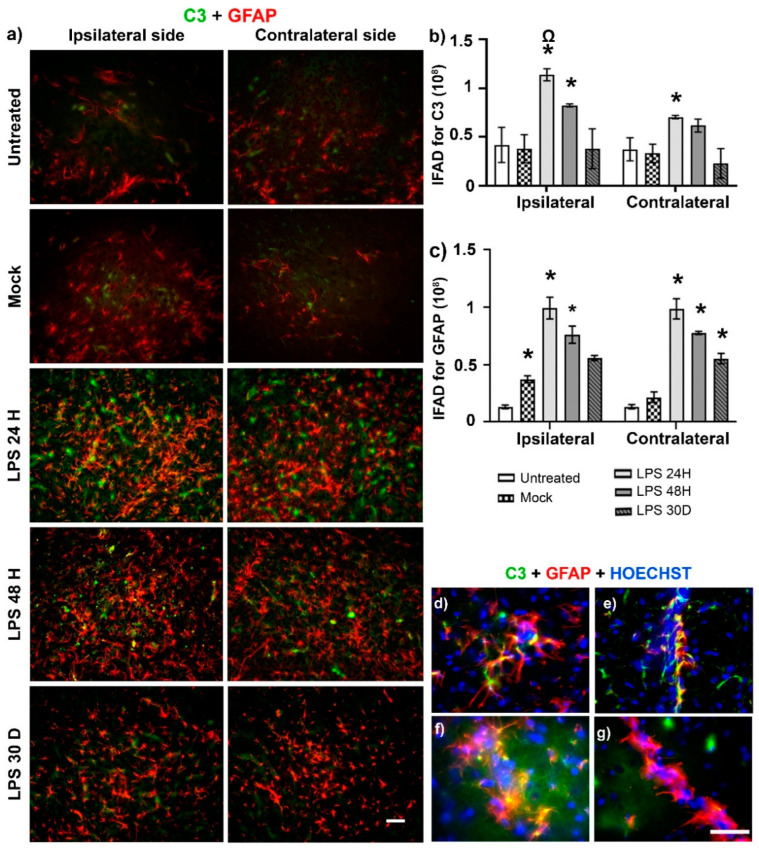
LPS induces the neurotoxic A1 phenotype in astrocytes of the SNpc. (**a**) Representative micrographs of double immunofluorescence against C3 (green) and GFAP (red) at different times (shown at the left margin in each row) post-LPS intranigral injection. The graphs show the IFAD for C3 (**b**) and GFAP (**c**) measured on the micrographs (**a**) using ImageJ software v.1.46r (National Institutes of Health; Bethesda, MD, USA). The bars represent the mean ± SEM calculated from the measurements in three anatomical levels per rat (*n* = 3 independent rats per group). Asterisks (*) denote statistically significant differences between the LPS groups with the Mock group on indicated sides of the brain. The omega symbol (Ω) compares the effect on the ipsilateral SN with that on the contralateral side. Two-way ANOVA and post hoc Tukey tests, *p* < 0.05. Representative amplified micrographs show details of neurotoxic A1 astrocytes in the ipsilateral (**d**,**e**) and contralateral sides (**f**,**g**) at 24 h post-LPS injection. The scale bars = 50 µm are for all micrographs.

Double immunofluorescence assays showed the induction of the neurotoxic A1 phenotype through C3 immunoreactivity in glial fibrillary acidic protein (GFAP) (+) cells [7] after LPS intranigral injection. The significant increase in C3 immunoreactivity (*p* < 0.05) followed the rise of GFAP (+) cells, expressed as immunofluorescence area density (IFAD), in the ipsilateral SN compared with the control groups and was 300% for C3 and 266% for GFAP at 24 h, 217% for C3 and 204% for GFAP at 48 h (Figure 3a,b). In the unlesioned SN (contralateral), LPS also significantly (*p* < 0.05) increased by 212% for C3 and 455% for GFAP at 24 h, and 186% for C3 and 358% for GFAP at 48 h (Figure 3a–c). The induction of C3 on the contralateral side was, however, 38% less than on the ipsilateral side at 24 h (*p* < 0.05) after LPS administration (Figure 3a,b). C3-immunoreactivity in the control groups was unspecific because it did not colocalize with GFAP (+) cells (Figure 3a). In contrast, the immunoreactivity of neurotoxic A1 astrocytes colocalized with the soma and processes of astrocytes and the astrocytic end-feet of the brain-blood-brain (BBB) vessels. The colocalization pattern was more evident on the ipsilateral than the contralateral side (Figure 3d–g).

**Figure 4:**
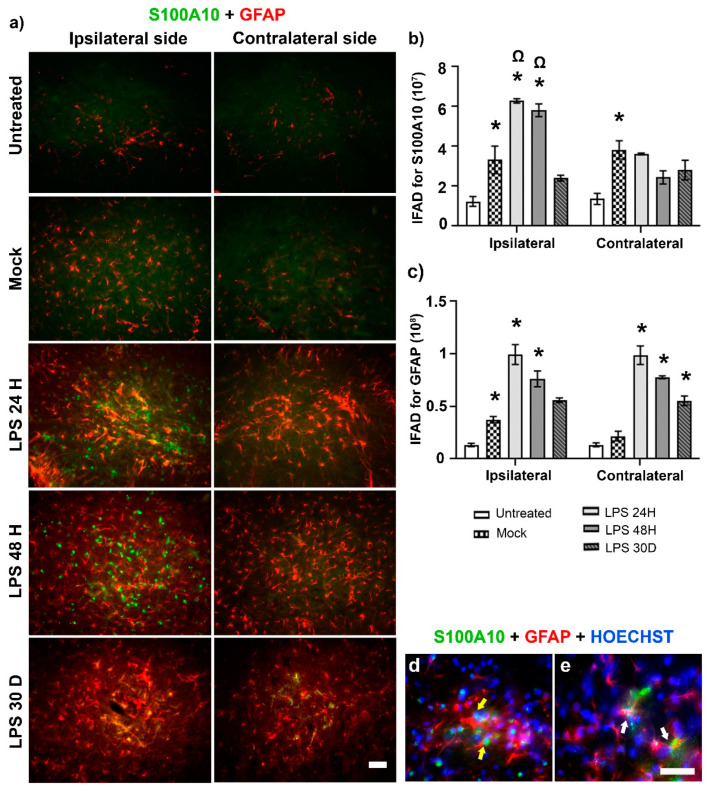
LPS induces the neurotrophic A2 phenotype in astrocytes of the SNpc. (**a**) Representative micrographs of the double immunofluorescence against S100A10 (green) and GFAP (red) at different times (shown at the left margin in each row) after LPS intranigral injection. The graphs show the IFAD for S100A10 (**b**) and GFAP (**c**) measured on the micrographs (**a**) using ImageJ software v.1.46r (National Institutes of Health; Bethesda, MD, USA). The bars represent the mean ± SEM calculated from the measurements in three anatomical levels per rat (*n* = 3 independent rats per group). The asterisk (*) compares the LPS group with the Mock group on both sides. The omega symbol (Ω) compares the LPS effect on the ipsilateral SN with that on the contralateral side at 24 h and 48 h. Two-way ANOVA and post hoc Tukey tests, *p* < 0.05. Representative amplified micrographs show S100A10 immunoreactivity in (**d**) nuclei (yellow arrows) of GFAP (+) cells in the ipsilateral SNpc and (**e**) the cytoplasm of astrocytes (white arrows) in the contralateral side at 24 h post-lesion The scale bars = 50 μm are for all micrographs.

LPS administration in vitro also induces the neurotrophic A2 phenotype in astrocytes identified by the S100A10 marker. Here, we found in vivo that the vehicle administration alone increased by 268% increase (*p* < 0.05) A2 immunoreactivity in GFAP (+) cells in the SN of both sides of the Mock group compared with the Ut control group, suggesting a defensive response of the brain to the mechanic injury caused by the injection procedure (Figure 4a–c). Furthermore, LPS administration produced an additional increase (*p* < 0.05), 189% at 24 h and 174% at 48 h, in A2 and GFAP immunoreactivities over those produced by the vehicle injection (Figure 4a–c). Although a significant increase in A2 (+) cells was detected in the contralateral SN at 24 and 48 h, that was 42% (24 h) and 58% (48 h) less than on the ipsilateral side (Figure 4a–c). Interestingly, A2 immunoreactivity displayed a different pattern in GFAP (+) cells depending on which side of the SN was evaluated; on the ipsilateral side, S100A10 staining was predominantly nuclear, whereas it was cytoplasmatic on the contralateral side (Figure 4d,e).

### 2.4. Inflammasome Activation in the SNpc following LPS Injection

**Figure 5:**
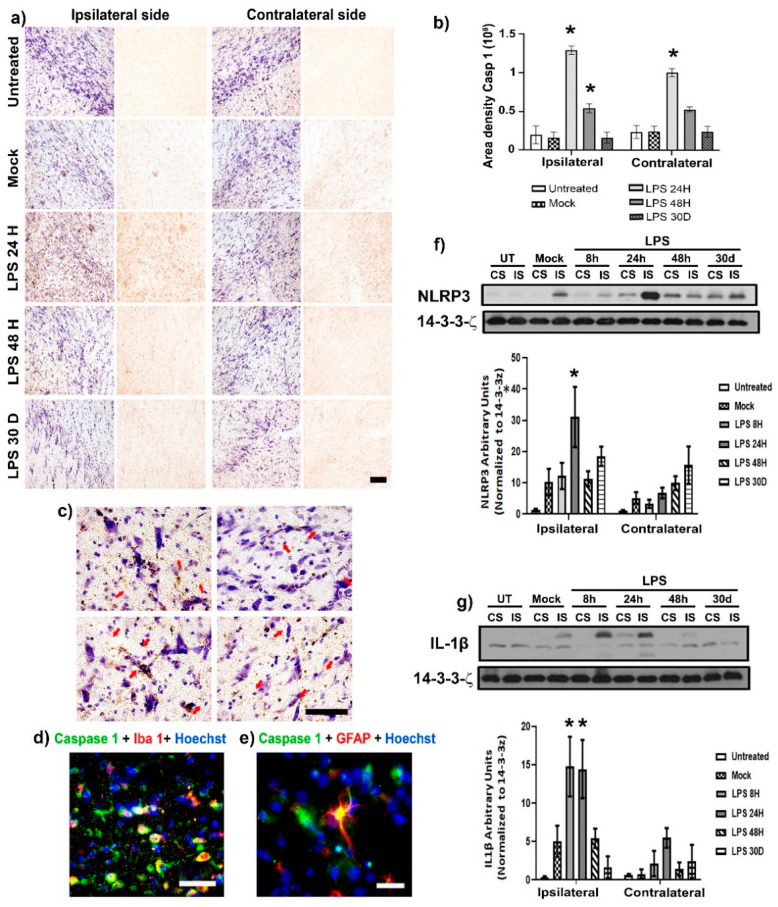
LPS caused the presence of active caspase-1 in microglia and astrocytes of the SNpc. (**a**) Representative micrographs of active caspase 1 (+) cells and Nissl counterstain in the SNpc of control groups (Ut and Mock) and groups at different times post-LPS injection (shown at the left margin in every row). (**b**) The graph shows the area density for active caspase-1 immunoreactivity quantified on the micrographs (**a**). (**c**) Representative micrographs of immunohistochemistry and Nissl staining suggest the presence of caspase-1 immunoreactivity on non-neuronal cells (red arrows). Double immunofluorescences with Hoechst counterstaining (blue) confirm caspase-1 immunoreactivity (green) in (**d**) microglia (red) and (**e**) astrocytes (red). The scale bars = 50 μm are for all micrographs. Quantification of NLRP3 (**f**) and IL-1β (**g**) in injured (IS) and contralateral side (CS) were measured by WB assays. The bars represent the mean ± SEM calculated from the measurements in three anatomical levels (*n* = 3 independent rats per group). The asterisk (*) compares the LPS groups with the control groups on both sides. Two-way ANOVA and post hoc Tukey tests, *p* < 0.05.

Since inflammasome activation plays a central role during the inflammatory response [33,34], its activation was evaluated using immunohistochemistry and western blot (WB) assays after an LPS intranigral injection. LPS injection significantly increased (*p* < 0.05) active caspase-1 immunoreactivity in the ipsilateral SNpc at 24 h (792%) and 48 h (333%) compared with the two control groups (Figure 5a,b). A significant 415% increase (*p* > 0.05) was also observed in the contralateral SNpc, but only at 24 h after LPS administration (Figure 5a,b). Detailed analysis through brightfield (Figure 5c) and fluorescent microscopy showed activated caspase-1 immunoreactivity in microglial cells (Figure 5b,d) and astrocytes (Figure 5e). Furthermore, WB analysis showed a significant 303.69% increase (*p* < 0.05) in +NLRP3 band density of the ipsilateral SN at 24 h after LPS injection when compared to the basal levels of Mock rats (Figure 5f). From the same samples, IL-1β band density also increased at 8 h (292.51%) and 24 h (286.25%) after LPS injection compared with the respective control values (*p* < 0.05; Figure 5g). No statistical significance (*p* > 0.05) was found in the contralateral groups.

### 2.5. LPS Administration Leads to a Decrease in the Mitochondrial CI Activity in SN Homogenates

**Figure 6:**
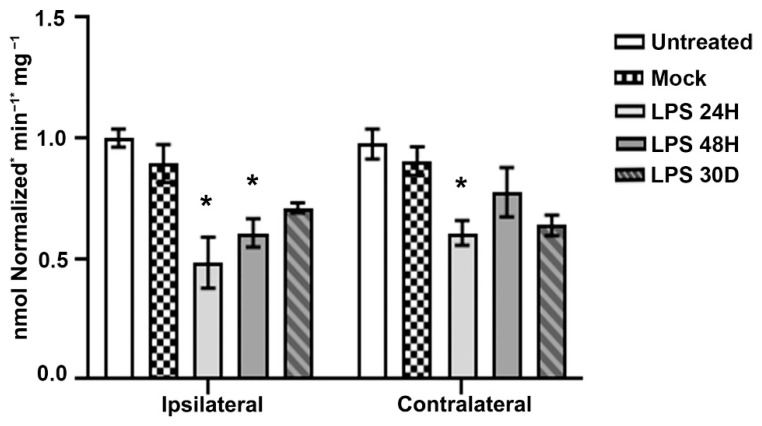
The intranigral LPS administration bilaterally decreases the mitochondrial complex I (CI) activity in homogenates of the entire substantia nigra. Data are expressed as the mean ± SEM (*n* = 6 independent rats per time point and experimental condition). The asterisk (*) compares LPS groups with the Mock and Ut groups. Two-way ANOVA and post hoc Tukey tests, *p* < 0.05.

A significantly decreased activity of mitochondrial complex I (CI) was found in both the ipsilateral (53.9%; *p* < 0.05) and contralateral SN (66.6%; *p* < 0.05) at 24 h after LPS administration compared with the Mock group (Figure 6). At 48 h, the decrease (60.7%; *p* < 0.05) in mitochondrial CI activity remained only in the ipsilateral hemisphere compared to the Mock group (Figure 6). No significant difference was found between control groups and LPS on day 30 after the LPS intranigral administration (*p* > 0.05; Figure 6) nor between the UT and Mock groups of both hemispheres (Figure 6).

### 2.6. LPS Induces Senescence in Dopaminergic Neurons

**Figure 7:**
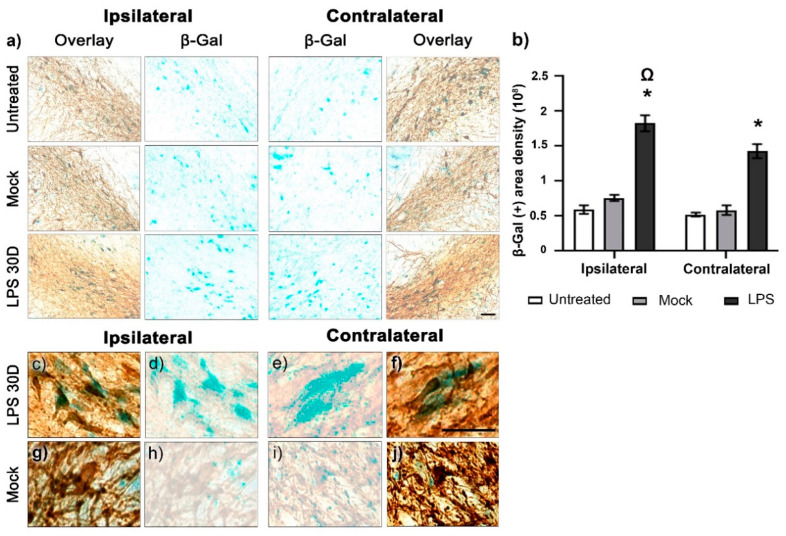
LPS induces senescence in dopaminergic neurons of the SNpc. (**a**) Representative micrographs of the SNpc with β-Gal staining and TH immunohistochemistry. (**b**) The Area density of β-Gal staining was measured by ImageJ software v.1.46r (National Institutes of Health, Bethesda, MD). The values are the mean ± SEM from three anatomical levels, *n* = 3 independent rats in each experimental condition. The asterisk (*) compares the LPS groups with the Mock group on both sides. The omega symbol (Ω) compares the ipsilateral SNpc with the contralateral side. One-way ANOVA and post hoc Tukey test, *p* < 0.05. Representative amplified photomicrographs show the collocation of β-Gal counterstaining with TH (+) cells in the SNpc of the LPS group (**c**–**f**) but not of the Mock group (**g**–**j**). The scale bars = 50 μm are for all micrographs.

β-Galactosidase (β-Gal) staining was used to evaluate whether LPS induced senescence in nigral dopaminergic neurons [32] 30 days after its injection. Compared to control groups, LPS-injected animals significantly increased (*p* < 0.05) the β-Gal area density by 240% on the ipsilateral SNpc and 243% on the contralateral side (Figure 7a,b). Statistical significance (*p* < 0.05) was found between the ipsilateral and contralateral sides on day 30 after LPS injection (Figure 7a,b). A detailed analysis showed that the β-Gal staining overlaps with TH (+) cells in the SNpc of both cerebral hemispheres (Figure 7c–f). In contrast, colocalization of these stainings was not detected in the control groups (Figure 7g–j).

### 2.7. Dopaminergic Nigrostriatal Neurodegeneration after LPS Intranigral Administration

**Figure 8:**
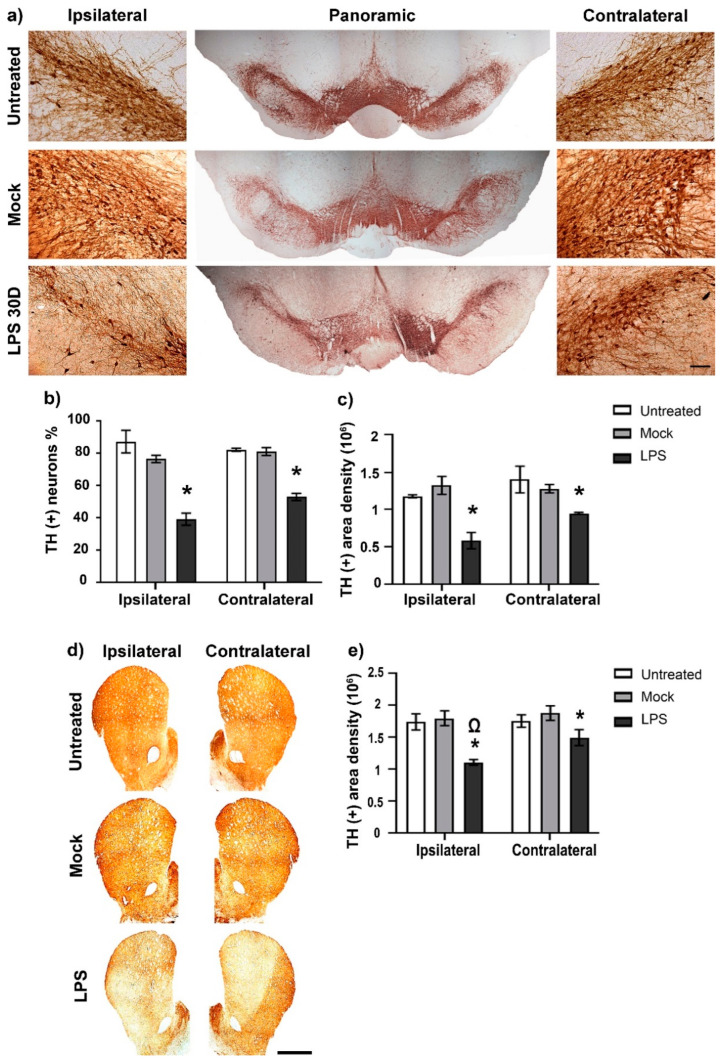
LPS intranigral injection decreases TH (+) cells and their ramification bilaterally in the SNpc and the striatum. (**a**) Representative micrographs of the mesencephalon processed with TH immunohistochemistry. Lateral panels show 20× amplification of the respective SNpc. (**b**) Neuron counting and (**c**) area density measurements in the substantia nigra. (**d**) Representative micrographs of the striatum processed with TH immunohistochemistry and (**e**) their area density measurements. ImageJ software v.1.46r (National Institutes of Health, Bethesda, MD) was used for quantifying neurons and area density of ramifications. The scale bar = 50 μm for micrographs of (**a**) and 1mm for micrographs of (**d**). The values are the mean ± SEM from three anatomical levels (*n* = 3 independent rats per experimental condition). The asterisk (*) compares LPS groups with the respective Mock group of both SN sides. The symbol (Ω) compares the LPS effect on the ipsilateral with that on the contralateral side. One-way ANOVA and post hoc Tukey tests, *p* < 0.05.

We quantified the number of TH (+) neurons, their ramification area density in the SNpc/SNpr, and the ramification area density in the striatum on day 30 after LPS intranigral administration (Figure 8). The TH (+) neurons counted was 222 (mean of three anatomical levels) in the ipsilateral SNpc and 219 on the contralateral side of healthy animals. LPS caused a significant loss of TH (+) cells (48%) and ramifications (55%) compared with the respective control values (*p* < 0.05) (Figure 8b,c). A loss of TH (+) cells (34%) and their ramification (32%) also occurred in the contralateral SNpc, which was statistically significant compared with the control group values (*p* < 0.05). No statistical significance (*p* > 0.05) was found between the ipsilateral and contralateral groups.

Similar results were obtained in the TH (+) area density of the striatum on day 30 after LPS intranigral injection. Again, TH (+) area density was significantly reduced (*p* < 0.05) in the ipsilateral striatum (38%) and contralateral striatum (25%) (Figure 8d,e). In addition, a significant difference (*p* < 0.05) in TH immunoreactivity was observed between the striatum of the two hemispheres on day 30 after LPS administration (Figure 8d,e).

### 2.8. Motor Behavior and Sensorimotor Deficit on Day 30 after LPS Intranigral Injection

**Figure 9:**
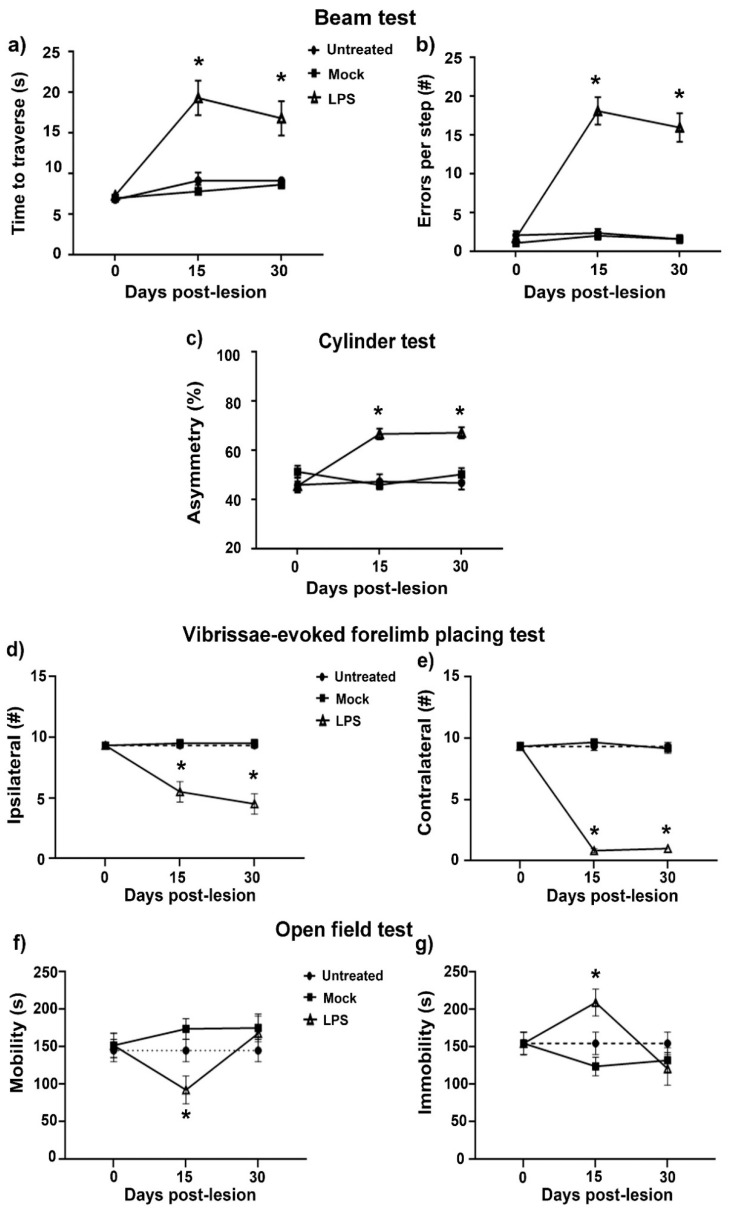
LPS intranigral injection elicits motor behavior and sensorimotor deficits on days 15 and 30 post-injection. Rats were evaluated with the beam test to measure (**a**) the travel time and (**b**) the number of errors per step. (**c**) The use of forelimbs was measured with the cylinder test to ascertain motor asymmetry. The sensorimotor deficit was assessed through the vibrissae-evoked response in the ipsilateral forelimb placing (**d**) and contralateral forelimb placing (**e**). Locomotor activity was quantified using the open field test by mobility time (**f**) and immobility time (**g**). Data are expressed as the mean ± SEM (*n* = 6 independent rats per time point and experimental condition). The asterisk (*) compares LPS groups with the Mock and Ut groups. One-way ANOVA and post hoc Tukey tests, *p* < 0.05.

Four independent motor behavior tests showed the physiological deficit of the LPS-induced dopaminergic nigrostriatal neurodegeneration [37]: the beam walking test to assess motor coordination, the balance and the limb-use asymmetry (“cylinder”) test to evaluate locomotor asymmetry, the vibrissae-evoked forelimb placing test to assess asymmetry in the sensorimotor cortex and striatum, and the open field test to analyze changes in locomotion. The evaluations were carried out in four groups of rats: (1) untreated, (2) Mock, and at (3) 15 and (4) 30 days after LPS administration (Figure 9).

The beam walking test revealed that the LPS injured rats spent a long time crossing the beam on days 15 (17.5 ± 6.5 s) and 30 (16.5 ± 6.5 s) compared with the Mock group (on days 15, 6.5 ± 2.5 s, and 30, 8.5 ± 1.5 s; both *p* < 0.05) and the Ut group (on day 15, 9.5 ± 3.5 s, and day 30, 9 ± 1 s). Besides, the injured rats made more errors during the stride on day 15 (15.5 ± 6.8 slips) and 30 (16 ± 6 slips) than the Mock group (on day 15, 2.5 ± 1.5 slips, and day 30, 2.5 ± 1.5 slips; *p* < 0.05) and Ut rats (on day 15, 3 ± 2 slips, and day 30, 2.5 ± 1.5 slips) (Figure 9b).

The cylinder test showed that LPS-injected rats exhibited locomotor asymmetry in the use of their forelimbs on days 15 (67.5 ± 7.5%) and 30 (69.4 ± 9.45%) compared with the Ut and Mock (*p* < 0.05) rats, which exhibited an equal use (50%) of their forelimbs (Figure 9c).

The vibrissae-evoked forelimb placing test showed that LPS caused a significant decrease (*p* < 0.05) in the response of ipsilateral forelimb placement on days 15 (5.5 ± 2.5) and 30 (4.5 ± 2.5), compared with the Mock group on day 15 (9 ± 1) and 30 (9.0 ± 1) (Figure 9d). LPS also significantly decreased (*p* < 0.05) the response of contralateral forelimb placements from days 15 (0.5 ± 0.5) and 30 (1.0 ± 0.5) after the lesion when compared with the Mock group on day 15 (9.5 ± 0.5) and day 30 (9 ± 1.0) (Figure 9e).

LPS caused a significant decrease in the mobility time of rats on day 15 after the LPS lesion (71.5 ± 51.5 s; *p* < 0.05; Figure 9f) when compared with the Mock group (172 ± 39 s). On that day, conversely, LPS significantly increased the immobility time of rats (232 ± 48 s; *p* < 0.05; Figure 9g) when compared with the Mock group (123 ± 34 s). In all four behavioral tests, the Mock group was not statistically different from the Ut group.

## 3. Discussion

This study confirms our results that a single administration of LPS in the SNpc triggers local acute neuroinflammation [29] and provides new findings consisting of the induction of neurotoxic A1 astrocytes, activation of NLRP3 inflammasome pathway, and mitochondrial impairment that, together with activated microglia, lead to neurodegeneration of the nigrostriatal system and sensorimotor deficits. Another novel finding is that neuroinflammation spread to the non-injected contralateral side, causing mitochondrial impairment and neurodegeneration of the nigrostriatal system. Our results further strengthen the contention that acute neuroinflammation can lead to neurodegeneration and help explain Parkinsonian variants independent of α-synucleinopathy [38].

We previously showed that microglia respond to the LPS stimulus as early as 0.2 h, releasing pro-inflammatory cytokines and acquiring a phagocytic shape [29]. In addition, we demonstrated that activated microglia and pro-inflammatory cytokine production precede reactive astrocytosis in the SN [29]. Therefore, the neurotoxic A1 astrocytes shown here could be induced by the activated microglia [5]. In agreement with previous reports [5,39], we also confirmed that LPS induced neurotrophic A2 astrocytes because they were double-positive to S100A10 and GFAP, suggesting the activation of anti-inflammatory mechanisms. Accordingly, after LPS stimulation, S100A10 protein in complex with the Ca^2+^/lipid-binding protein annexin A2 (annexin A2–S100A10 complex) [40] inhibits astrocytic proliferation and modulates inflammasome activation as well as cytokines released in the CNS [41]. However, the detrimental effect of LPS prevailed despite neurotrophic A2 astrocytes, as shown here and by others [14,28,42], thus indicating that the endogenous neurotrophic mechanism needs further reinforcement to overcome the neurotoxic effect of A1 astrocytes [16,43,44].

Microglial NLRP3 inflammasome has recently been shown to play a critical role in the induction of neurotoxic reactive astrocytes A1 through the release of a cytokine cocktail involving the upstream NF-kB pathway and the downstream activation of caspase-1 [45]. Accordingly, LPS induced the activation of NLRP3 inflammasome and caspase-1 and its subsequent processing of IL-1β only in the ipsilateral SN, thus accounting for neurotoxic reactive astrocyte A1 activation. As a hypothesis to explain the dopaminergic neurodegeneration on the contralateral side in the absence of the NLRP3 inflammasome activation, we propose that activated microglia cells increase the release of harmful immunomodulatory molecules to induce the neurotoxic reactive astrocytes A1 [46]. Microglia could likely be the most plausible candidate cells for spreading neuroinflammation on the contralateral side because these cells are highly dynamic and scan the entire brain in a short time [47,48]. Therefore, the neurodegeneration of the contralateral nigrostriatal system found on day 30 after LPS administration could result from the direct action of neurotoxic reactive astrocytes A1, activated microglia, and the poor neurotrophic contribution of reactive astrocytes A2.

Increasing evidence also supports the pathological role of NLRP3 inflammasome in neurodegeneration based on a mutual association with mitochondrial dysfunction [36,49,50,51], which correlates with neurodegeneration in parkinsonian rats with α-synucleinopathy [52]. Accordingly, we have shown here that mitochondrial CI dysfunction and NLRP3 activation coincided in the early phase of LPS-induced neuroinflammation and with an increase in nitrosative/oxidative stress, as we previously demonstrated [29]. Our results suggest that all these early events can lead to dopaminergic nigrostriatal system neurodegeneration and sensorimotor deficit. Future research requires evaluating whether there also would be a mitochondrial metabolism impairment and identifying the neuronal lineage affected.

Dopaminergic neurodegeneration was proposed based on the reduction of TH (+) cells in the SNpc found on day seven after LPS-induced neuroinflammation [29]. Here, we showed that it remained until 30 days and also occurred in the contralateral control SNpc and both striata. Furthermore, the remaining nigral TH (+) cells of both hemispheres were also positive for β-Gal, an enzymatic product of the senescence-associated β-Galactosidase (SA-β-gal) [53,54], thus showing senescent dopaminergic neurons. Given that mitochondrial dysfunction triggers neuronal senescence via oxidative stress [55,56], we think the impairment of mitochondria might be involved in the LPS-induced senescence of dopaminergic neurons. This suggestion is further supported by the finding that LPS systemically administered triggers mitochondrial impairment associated with injury in dopaminergic neurons and sensorimotor alterations [57]. However, colocalization studies must be performed to demonstrate soundly that mitochondrial impairment and oxidative stress occur in senescent dopaminergic neurons.

Furthermore, it has been shown that A1 astrocytes can also disrupt the BBB by releasing inflammatory mediators, including TNF-α and several chemokines [58], which promote leukocyte infiltration [29,59]. Here, we documented that the neurotoxic A1 astrocytic end-feet immunoreactivity is more evident on the ipsilateral than the contralateral side, suggesting a BBB disruption predominantly on the ipsilateral side. This dysbalance can account for the predominance of neurodegeneration in the ipsilateral nigrostriatal system. Therefore, ameliorating astrocytic dysfunction is a promising therapeutic approach for modulating BBB functions and treating neurological diseases [58]. One strategy aims to block the microglia-mediated transformation of astrocytes to an A1 phenotype using the glucagon-like peptide-1 receptor agonist (for which NLY01 is under clinical investigation) [28]. Another consists of mesenchymal stem cell-derived exosomes to suppress A1 activation by downregulating the NF-p65 phosphorylation subunit, as shown in spinal cord injury [60,61]. Conversely, excitation of specific signaling pathways in A2 astrocytes can improve cognitive impairment; for example, insulin therapy was shown to enhance the mental competence of patients with Alzheimer’s disease due to insulin receptor activation on A2 astrocytes [62,63]. Besides, serotonin 1A (5-HT1A) receptors accelerate the expression of antioxidative molecules by A2 astrocytes in a PD mouse model, and the 5-HT1A agonist 8-OH-DPAT can protect dopaminergic neurons [64]. All these findings suggest that shifting astrocytes from an A1 to A2 phenotype can potentially treat neurodegenerative diseases.

In conclusion, our results show that LPS induces acute neuroinflammation associated with activated microglia and the predominant participation of reactive astrocytes A1 versus A2 phenotype, NLRP3 inflammasome pathway activation, and mitochondrial CI dysfunction. All these events may lead to neuronal senescence and dopaminergic nigrostriatal neurodegeneration. Therefore, the intranigral administration of LPS is a suitable model of parkinsonism caused by endotoxins that can also help assess new antiparkinsonian treatments.

## 4. Materials and Methods

### 4.1. Experimental Animals

Two-month-old male Wistar rats (200–210 g) were used according to protocol #162-15, authorized and supervised by the Institutional Committee for the Care and Use of Laboratory Animals of the Center for Research and Advanced Studies (CINVESTAV) following the current Mexican legislation, NOM-062-ZOO-1999, and NOM-087-ECOL-1995 (Secretaría de Agricultura, Ganadería, Desarrollo Rural, Pesca y Alimentación; SAGARPA). Considering the three R’s (Reduction, Refinement, and Replacement) for animal experimentation [65], the number of animals was kept to a minimum according to the experimental design and statistical significance (The National Academies Collection: Reports funded by National Institutes of Health, 2011). Moreover, all efforts were made to minimize animal suffering.

Five rats were housed per acrylic cage (34 cm × 44 cm × 20 cm) in a room with controlled conditions of temperature (22 ± 2 °C), relative humidity (60 ± 5%), inverted light-dark 12 h cycles, and food and water ad libitum. Ninety-three animals were randomly distributed into three experimental groups (Supplementary Figure S1): (1) Untreated (Ut) rats with no procedure, (2) Mock rats with an intranigral injection of endotoxin-free sterile physiological saline solution, the vehicle, (3) LPS rats with an intranigral injection of the endotoxin solution. Eighteen rats were for the time course evaluation of sickness behavior and temperature until the first 24 h post-lesion (*n* = 6 per group). Eighteen rats were evaluated with two sensorimotor tests before surgery, on day 15 and day 30 post-lesion (*n* = 6 per group; euthanized on day 30). Some rats were euthanized at 24 h (the animals used in the sickness behavior), 48 h (*n* = 3 per experimental group), and day 30 (the animals used in the sensorimotor tests) for immunostaining studies. The mitochondrial CI activity assays were analyzed simultaneously with the histology studies (*n* = 6 per group). Finally, the western blot tests (*n* = 3 per group) were evaluated at 8 h, 24 h, 28 h, and 30 days post-LPS administration.

### 4.2. Stereotaxic Procedure

Animals were anesthetized with a single injection of a mixture of ketamine (70 mg/kg) and xylazine (6 mg/kg) via intraperitoneal (i.p.) and fixed in a stereotaxic apparatus (Stoelting; Wood Dale, IL, USA). The LPS used in all experiments was from *Escherichia coli* serotype 055: B5 (Sigma-Aldrich; St. Louis, MO, USA). A single dose of LPS (5 μg/2 μL of endotoxin-free physiological saline solution) was injected into the left SN in the following coordinates: anteroposterior (AP), +3.2 mm from the interaural midpoint; mediolateral (ML), +2.0 mm from the intraparietal suture; and dorsoventral (DV), −6.5 mm from the dura mater [29]. The Mock group was injected with 2 μL of endotoxin-free physiological saline at those coordinates. The solutions were injected at 0.13 μL/min with a microperfusion pump (Stoelting; Wood Dale, IL, USA), so the total injection time was 15.4 min. After the total dose was injected, the needle was allowed to remain in the brain for 5 min and then withdrawn in 1-min steps.

### 4.3. Body Surface Temperature

A hand-held infrared thermometer (DT-8809CC; Pioway Medical; Shenzen, China) was used to measure the temperature on the shaved dorsum of the animal body. The measurements were recorded in a temporal course (1, 2, 3, 4, 5, 6, 7, 8, 21, and 24 h) before and after the intranigral injection of LPS or endotoxin-free physiological saline solution.

### 4.4. Sickness Behavior

Sickness behavior consists of behavioral signs in sick individuals developed during an infection. The usually evaluated LPS-elicited signs are lack of exploration and locomotion, curled body posture, irregular fur, piloerection, and closed eyes. [66]. Measurements were performed over time (1, 2, 3, 4, 5, 6, 7, 8, 21, and 24 h) in rats kept in transparent cages and scored on a four-point scale: 0 = no signs, 1 = one sign, 2 = two signs, and 3 = three or more signs after the intranigral injection of LPS [29].

#### 4.4.1. Beam Walking Test

The beam walking test evaluated the balance, motor coordination, and traveled time while rodents crossed a narrow beam (1 cm wide and 2 m long, placed at 45° tilt) [37]. The test was carried out after two days of pre-training. The final test was videotaped on the third day. Then, the number of errors per step of the hind limbs (slips) and the traveled time were measured on videos. The number of slips is a more exact measure of the motor deficit during gait on a beam [32].

#### 4.4.2. Limb-Use Asymmetry (“Cylinder”) Test

The cylinder test evaluated the locomotor asymmetry of the forelimbs when they touched the walls of a transparent acrylic cylinder (30 cm tall, 20 cm diameter) [37]. First, the behavior was video recorded to evaluate the first 20 contacts made with the ipsilateral, the contralateral, and both paws simultaneously [67]. The lesioned side is the reference to refer to the ipsilateral and contralateral contacts. Then, the percentage of locomotor asymmetry was calculated with the following formula:

$$\%\;\mathrm{of}\;\mathrm{asymmetry}\;=\frac{\mathrm{contacts}\;\mathrm{of}\;\mathrm{ipsilateral}\;\mathrm{forelimb}+\frac{1}{2}\;\mathrm{simultaneous}\;\mathrm{contacts}}{\mathrm{ipsilateral}+\mathrm{contralateral}+\mathrm{simultaneous}\;\mathrm{contacts}}\times 100$$

Previous to the test, the cylinder was cleaned with 30% ethanol.

#### 4.4.3. Vibrissae-Evoked Forelimb Placing Test

This behavioral test shows the asymmetry in the sensorimotor cortex [37,67]. Animals were gently held through the torso so that all four limbs hung freely. The vibrissae of each side were stimulated by brushing against the edge of a table to elicit an ipsilateral forelimb response that consisted in placing the paw on the tabletop. The expected reaction of healthy animals includes a quick placement of their forelimb on the table surface. Its deficit primarily reveals a motor alteration rather than a sensory one. The rat was allowed ten trials on the contralateral and ipsilateral sides, and the percentage of successful placements were recorded.

#### 4.4.4. Open Field Test

The open-field test evaluates the exploration behavior of a new environment through spontaneous locomotor activity [37,67]. In a quiet room, the rats were placed in the center of a square polyvinyl chloride (PVC) box (80 × 80 cm) 40 cm high. A camera was used to monitor walking and immobility times for 10 min. The arena was cleaned with a water/alcohol (30%) solution before every behavioral test to avoid a possible bias due to odors and residues left by rats tested earlier.

### 4.5. Immunostaining Procedures

Rats were anesthetized with sodium pentobarbital (50 mg/kg i.p.) and perfused through the ascending aorta with 30 mL of phosphate-buffered saline (PBS), followed by 90 mL of 4% paraformaldehyde in PBS. Their brains were removed and post-fixed in 4% paraformaldehyde for 24 h, followed by cryoprotection in 30% sucrose. Serial coronal sections of 30 μm thickness were cut using a sliding microtome with a freezing stage (Leica SM1100; Heidelberg, Germany), collected in 6 wells, and only the slices in one well were analyzed. The slices were permeabilized with PBS-0.1% Triton for 20 min and incubated with bovine serum albumin (BSA) in PBS-0.1% Triton for 30 min to block nonspecific binding sites at room temperature (RT).

#### 4.5.1. Immunohistochemistry Assays

After blocking unspecific binding sites, the endogenous peroxidase activity was quenched using a solution of 3% hydrogen peroxide/10% methanol in PBS for 30 min at RT. Then, the slices were incubated with the proper primary antibody for 24 h at 4 °C. The primary antibodies were mouse monoclonal anti-TH (1:1000; Sigma-Aldrich; St. Louis, MO, USA), goat polyclonal anti-Iba1 (1:1000; Abcam; Cambridge, UK), and rabbit polyclonal anti-cleaved caspase 1 (1: 200; Invitrogen Molecular Probes; Eugene, OR, USA). After removing the primary antibody and washing with PBS, suitable secondary antibodies were incubated for 2 h at RT and were biotinylated horse anti-mouse IgG (1:300; Vector Laboratories; Burlingame, CA, USA), biotinylated horse anti-goat IgG (1:300; Vector Laboratories; Burlingame, CA, USA), and biotinylated donkey anti-rabbit IgG (1:500; Zymed; Cambridge, MA, USA). Subsequently, the tissues were incubated with avidin-biotin-peroxidase complex (ABC Kit; Vector Laboratories; Burlingame, CA, USA), and color was produced with 3,3-diaminobenzidine (DAB; Sigma-Aldrich; St. Louis, MO, USA). In addition, some tissues were counterstained with β-Gal to evidence cellular senescence [68] and Nissl (0.1% cresyl violet) to specifically stain the cytoplasm of the neurons and so delimit the SN [32,69].

Finally, the brain slices were washed 3 times for 5 min in PBS, mounted on slides using Entellan resin (Merck, KGaA; Darmstadt, Germany), and observed with a light Leica DMIRE2 microscope equipped with 5×, 20×, 40×, and 63× (oil immersion) objectives (Leica Microsystems; Nussloch, Germany). The images were observed through light-field with a Leica DMIRE2 microscope (Leica; Nussloch, Germany) and digitalized with a Leica DC300F camera (Leica Microsystems; Nussloch, Germany).

#### 4.5.2. Immunofluorescence Assay

Immunofluorescence assays were performed after blocking nonspecific binding sites [7]. For double immunofluorescence assays, the samples were incubated with the suitable pair of primary antibodies (Supplementary Table S1) at 4 °C overnight. The primary antibodies were rabbit polyclonal anti-complement C3 (1:50; Abcam; Cambridge, UK), mouse monoclonal anti-GFAP (1:500; Cell Signaling Technology; Danvers, MA, USA), rabbit polyclonal anti-S100A10 (1:100; Invitrogen Molecular Probes; Eugene, OR, USA), rabbit polyclonal anti-cleaved caspase 1 (1:200; Invitrogen Molecular Probes; Eugene, OR, USA), and goat polyclonal anti-Iba1 (1:1000; Abcam; Cambridge, UK). After incubation, the tissues were washed and incubated with suitable sets of secondary antibodies (Table S1) for 2 h at RT. The secondary antibodies were Alexa Fluor 488 chicken anti-rabbit H+L IgG (1:300; Invitrogen Molecular Probes; Eugene, OR, USA), Texas red horse anti-mouse H+L IgG (1: 900; Vector Laboratories; Burlingame, CA, USA), and Alexa Fluor 555 donkey anti-goat H+L IgG (1:300; Abcam; Cambridge, MA, USA). For nuclear counterstaining, some brain slices were incubated with 1 μM Hoechst dye (Sigma-Aldrich; St. Louis, MO, USA) for 5 min following immunostaining. After washing with PBS, the slices were mounted on glass slides using VECTASHIELD (Vector Laboratories; Burlingame, CA, USA). Fluorescence images were obtained with a Leica DMIRE2 microscope (Leica; Nussloch, Germany) through filters A for Hoechst dye, K3 for Alexa Fluor 488 (green fluorescence), and TX2 for Texas red and Alexa Fluor 555 (red fluorescence) using 20× and 63× objectives. Images were digitalized with a Leica DC300F camera (Leica Microsystems; Nussloch, Germany).

### 4.6. Densitometry and Neuron Counting

ImageJ software (National Institutes of Health; Bethesda, MD, USA) was used to count TH(+) neurons in the SN and measure the total area density of TH(+) nigral ramifications and striatal terminals on immunohistochemistry stained slices [67]. The measurement was made with images taken with the objectives 20× for the SNpc and SNpr and 5x for the striatum. All background intensity was eliminated from the immunohistochemically stained area to quantify only TH(+) immunoreactivity. The neuron counting and area density of TH(+) ramifications were determined at least at three anatomic levels (*n* = 3 rats for each experimental group), as we reported previously [7,29]. The final measurement was the mean value calculated from the quantification in the three levels per nucleus and per rat. A similar procedure was followed to measure the area density of β-Gal staining, Iba 1 (microglial cells maker), and caspase 1 in the SN. We used the following morphological criteria to discern the physiological state of microglia: Quiescent microglial cells (normal physiological condition) present a highly branched morphology with small nuclei and soma, while active microglial cells transform into almost spherical cells (amoeboid) with prominent soma, disappearing the longest dendritic extensions [70,71].

Fiji, an Image J complement, was used for color decomposition from the double staining of β-Gal staining with TH and cresyl violet staining with Iba1/caspase-1 [32].

The IFAD for the double fluorescence assays was measured by ImageJ software (The National Institutes of Health; Bethesda, MD, USA) in three anatomic levels of the SN per rat (*n* = 3 independent rats per experimental condition). The final measurement was the mean value calculated from the quantification in the three levels per nucleus and per rat.

### 4.7. Western Blot Assay

IL-1β and NLRP3 were detected by western blot following the procedure reported previously [72]. Proteins from samples of SN were extracted in radioimmunoprecipitation assay buffer (150 mmol/L NaCl, 1% NP-40, 0.5% deoxycholic acid, 0.1% SDS, and 50 mmol/L Tris, pH 8.0) and homogenized using a Benchmark BeadBlaster Homogenizer (Benchmark Scientific; Sayreville, NJ, USA). Protein concentration was determined using the BCA Protein Assay (Pierce, Thermo Fisher Scientific; Waltham, MA, USA). A total of 25 µg of protein was loaded for Western blot analysis. After blocking in 5% skim milk in 50 mL of PBS (8.1 mM Na_2_HPO_4_, 1.2 mM KH_2_PO_4_, 138 mM NaCl, 2.7 mM KCl, pH 7.4)—0.1% Tween 20 for 1 h with shaking, membranes were incubated with either a rabbit polyclonal antibody to IL-1β (1:1000 dilution; Abcam; Cambridge, UK) and a rabbit polyclonal antibody to NLRP3 (1:1000 dilution; Cell Signaling Technology Inc., Danvers, MA, USA)at 4 °C overnight. Secondary antibodies (incubation 2 h at room temperature) were a peroxidase-labeled donkey anti-rabbit IgG (1:10,000 dilution; Amersham Bio-Sciences; Piscataway, NJ, USA. To normalize the total amount of protein per lane, membranes were stripped and incubated with a rabbit polyclonal antibody against 14-3-3-z (1:1500 dilution; Santa Cruz Biotechnology) and the peroxide-labeled goat anti-rabbit IgG (1:10,000 dilution; Abcam; Cambridge, UK). The immunoreactivity was detected by chemiluminescence.

### 4.8. Mitochondrial Complex I (CI) Activity Determination in the Substantia Nigra Homogenate

The mitochondrial CI activity was determined as reported elsewhere [52]. The tissue from an entire SN was homogenized in PBS pH 7.4 and subsequently centrifugated for 5 min at 2000 g. The supernatants were collected and used to perform the analyses. CI activity measurement is based on its ability to oxidize nicotinamide adenine dinucleotide + hydrogen (NADH) while reducing decylubiquinone (Dub) to dihydro-decylubiquinone (DUbH2), a molecule that is next oxidized by 2,6-Dichloroindophenol (DCPIP). This product can absorb light at 600 nm. In the meantime, 20 mM rotenone was included as a specific inhibitor of CI. All the measurements were completed employing a Synergy-Biotek (Biotek Instruments; Winooski, VT, USA) microplate reader at 37 °C. The CI activity was defined by subtracting the activity obtained with the inhibitor rotenone from the not inhibited activity and expressed as nmol/min/mg protein (data normalized by mean values of the not treated group).

### 4.9. Statistical Analysis

All results were expressed as the mean value ± the SEM at least from 3 independent experiments (*n* = 3). Intergroup differences were evaluated by bidirectional analysis of variance (2-way ANOVA), followed by the Tukey post hoc test. GraphPad Prism 5.0 software (GraphPad Software Inc., La Jolla, CA, USA) was used for statistical analysis. Statistical difference was considered at *p* < 0.05.

## Data Availability

The data that support the findings of this study are available from the corresponding author upon reasonable request.

## Supplementary Materials

The following supporting information can be downloaded at: https://www.mdpi.com/article/10.3390/ijms24054628/s1.
