# Supplementary material for: LPS Triggers Acute Neuroinflammation and Parkinsonism Involving NLRP3 Inflammasome Pathway and Mitochondrial CI Dysfunction in the Rat
Source: Int J Mol Sci. 2023 Feb 27;24(5):4628. doi: 10.3390/ijms24054628 (PMC10003606; doi:10.3390/ijms24054628)
Supplement: Supplementary file 1 [file ijms-24-04628-s001.zip › ijms-1836553-supplementary.pdf]

a) Experimental design

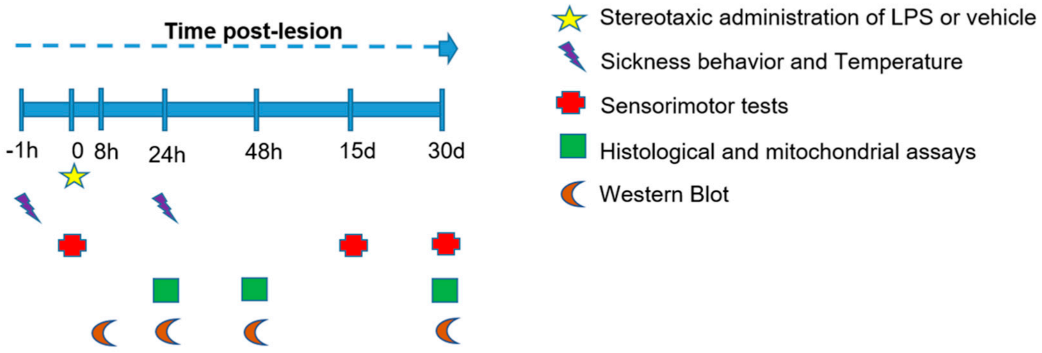

b) Numbers of animals

Per experiment		Group	Per times	Subtotal
Sickness behavior and temperature	*n* = 6	Untreated	X 1	*n* = 6
		Mock	X 1	*n* = 6
		LPS	X 1	*n* = 6
Sensorimotor test	*n* = 6	Untreated	X 1	*n* = 6
		Mock	X 1	*n* = 6
		LPS	X 1	*n* = 6
Immunostaining	*n* = 3	Untreated	X 1	*n* = 3
		Mock	X 1	*n* = 3
		LPS	X 1	*n* = 3
Western Blot	*n* = 3	Untreated	X 1	*n* = 3
		Mock	X 1	*n* = 3
		LPS	X 4	*n* = 12
Mitochondrial assay	*n* = 6	Untreated	X 1	*n* = 6
		Mock	X 1	*n* = 6
		LPS	X 3	*n* = 18
				**Total = 93**

Supplementary Figure S1. Experimental design. (**a**) Color symbols indicate when the corresponding assays were performed. (**b**) The table summarizes the number of animals used per experiment at every time and group evaluated. The immunostainings were performed at 24 h (animals used in the sickness behavior), 48 h, and 30 d (the animals used in the sensorimotor tests).

Table S1. Antibody combination for double immunofluorescence.

Epitope	Primary Antibodies	Secondary Antibodies	Objective
C3	rabbit polyclonal anti-complement C3	Alexa Fluor 488 chicken anti-rabbit H+L IgG	Neurotoxic A1 astrocytes
GFAP	mouse monoclonal anti-GFAP	Texas red horse anti-mouse H+L IgG	
S100A10	rabbit polyclonal S100A10	Alexa Fluor 488 chicken anti-rabbit H+L IgG	Neuroprotective A2 astrocytes
GFAP	mouse monoclonal anti-GFAP	Texas red horse anti-mouse H+L IgG	
Caspase 1	rabbit polyclonal anti-caspase 1	Alexa Fluor 488 chicken anti-rabbit H+L IgG	Active caspase 1 in astrocytes
GFAP	mouse monoclonal anti-GFAP	Texas red horse anti-mouse H+L IgG	
Caspase 1	rabbit polyclonal anti-caspase 1	Alexa Fluor 488 chicken anti-rabbit H+L IgG	Active caspase 1 in microglial cells
Iba 1	goat polyclonal anti-Iba1	Alexa Fluor 555 donkey anti-goat H+L IgG	
